# Exploring the Longitudinal Relationship Between Short Sleep Duration, Temperament and Attention Deficit Hyperactivity Disorder Symptoms in a Biethnic Population of Children Aged Between 6 and 61 Months: A Born in Bradford Study

**DOI:** 10.1177/10870547231168433

**Published:** 2023-05-08

**Authors:** Jonathan Stott, Elizabeth Coleman, Anna Hamilton, Jane Blackwell, Helen L. Ball

**Affiliations:** 1 Leeds and York Partnership NHS Foundation Trust, UK; 2 Tees, Esk and Wear Valleys NHS Foundation Trust, UK; 3 Hull York Medical School, UK; 4 University of York, North Yorkshire, UK; 5 University of Birmingham, UK; 6 Durham University, UK

**Keywords:** parent-reported sleep duration, ADHD, ethnic group, pediatrics, Born in Bradford

## Abstract

**Objective:**

Examine the association between sleep duration, temperament and symptoms of Attention Deficit Hyperactivity Disorder (ADHD) in a biethnic child-population from The Born in Bradford cohort.

**Method:**

Parent-report sleep duration categorized children as: early short, late short, consistently short or consistently normal sleepers between 6 and 36 months. Temperament was measured using the Infant Characteristics Questionnaire at 6 months. The Strengths and Difficulties Questionnaire assessed symptoms of ADHD at 37, 54, and 61 months.

**Results:**

Normal sleepers before 18 months had significantly fewer ADHD symptoms at 37 months compared with consistently short sleepers. Fussiness at 6 months was significantly positively associated with ADHD symptoms at 37 and 54 months; but does not appear to mediate the relationship between sleep duration and ADHD symptoms.

**Conclusion:**

Awareness of the relationship between short sleep duration and fussiness in infancy and later ADHD symptomatology may support earlier identification of arising difficulties in children.

## Introduction

Attention Deficit Hyperactivity Disorder (ADHD) is one of the most common neurodevelopmental conditions, affecting around 5-7% of school-aged children (Willcutt, 2012), and is more commonly diagnosed in boys than in girls (Nøvik et al., 2006). Onset of ADHD symptoms is typically in childhood (Efron, 2019) and referrals for assessment are often made when the child is between 6 and 7 years of age, when the developmentally inappropriate and poorly regulated activities impact the child’s daily functioning and their ability to keep up with their peers.

The estimated prevalence of ADHD in children aged 5 to 15 in the UK is 3.6% in males and 0.9% in females (Ford et al., 2003), which is lower than the worldwide estimates (Holden et al., 2013; Nøvik et al., 2006). Prevalence rates vary dramatically between countries and are highest in the USA at 8.1% and lowest in Iraq (0.1%), Poland (0.3%) and Romania (0.4%) (Fayyad et al., 2017). Rates of diagnosis and treatment also vary across different ethnicities; non-white young people are less likely to access healthcare services, be diagnosed, and receive treatment for ADHD than white young people (Chung et al., 2019; Fairman et al., 2020; Wright et al., 2015). In Pakistan, ADHD was one of the least commonly diagnosed psychiatric disorders among adults in tertiary hospitals (Khan et al., 2020) and Asian American patients are approximately 75% less likely to receive a diagnosis of ADHD than white patients (Chung et al., 2019).

Sleep problems are common in childhood ADHD, with prevalence rates ranging from 25% to 85%. (Owens, 2005; Spruyt & Gozal, 2011; Sung et al., 2008; Yoon et al., 2012; Yürümez & Kılıç, 2016). Short sleep duration is associated with ADHD symptomatology, especially hyperactive symptoms, as well as being a potential risk factor for ADHD (Lee et al., 2019). The impact of impaired sleep duration on later ADHD symptoms has been demonstrated from as early as 3 months of age (Huhdanpää et al., 2019).

Determination of ADHD at baseline is challenging. Previous research suggests that measures of negative emotionality may provide an index for early detection of ADHD (Sullivan et al., 2015). In addition, associations were found between infant temperament and sleep problems in children with ADHD at 5 years of age suggesting that temperament may be an endophenotype that underpins the relationship between ADHD and sleep disturbance (Melegari et al., 2020).

Regulatory problems such as excessive crying, sleeping difficulties and/or feeding problems are reported in approximately 20% of infants in the first year of life (DeGangi et al., 2000; Forsyth, 1991; Wolke et al., 1995) and are associated with difficulties with self-regulation of fussiness, irritability or coping with change (Zero to Three 2005). Evidence suggests that multiple regulatory problems can affect children’s long term behavior (Degangi et al., 1991; DeSantis et al., 2004; Scher, 2005; Wolke et al., 2002). Meta-analysis shows that children with previous regulatory problems have more behavioral problems, particularly ADHD behaviors, than those without, but more research is needed to investigate the behavioral outcomes of previously sleep-disturbed children (Hemmi et al., 2011).

Larger samples, prospective cohort studies and those including children demonstrating sleep-disturbance are needed to investigate these ideas further. To address the gap in the literature, the aims of the current study were to (a) identify whether sleep duration has an independent association with ADHD symptoms after controlling for potential confounders; (b) identity whether infant temperament at 6 months has an independent association with ADHD symptoms in early childhood; and (c) examine whether any association between sleep duration and ADHD symptoms is mediated through infant temperament. Based on existing literature, we anticipated that both sleep duration and infant temperament would be associated with ADHD symptoms and that parent-reported infant temperament mediates the association between sleep duration and ADHD symptoms.

### Methods

Within the BiB data set were 28 sets of twins which have not been excluded from the data set utilized here. The characteristics of their parents have been included as if they were separate entities. Fifty children were excluded from the analysis as their comorbidities, present at 6 months of age, are known to be associated with ADHD: Down’s syndrome (*n* = 3), reflux/milk allergy (*n* = 31), prematurity (*n* = 15), Canavan disease (*n* = 1), I-cell disease (*n* = 1) and sleep apnea (*n* = 1), where multiple reasons for exclusion were present in two children. Thus, in total 1,713 children and their parents were included within this study.

### Demographic Information

Baseline demographic data collected included ethnicity, gender, mother’s and father’s highest qualification and age.

#### Measures

Symptoms of inattention, impulsiveness, impulsiveness and hyperactivity were assessed at three separate timepoints; 37 and 54 months by the parent, and 61 months by a teacher, using the Strengths and Difficulties Questionnaire (SDQ). With an established factor structure, the SDQ has demonstrated utility as an ADHD disorder screening measure in the community (Algorta et al., 2016; Hall et al., 2019; Ullebø et al., 2011). It addresses four areas of potential difficulty. The outcome of interest was the five-item hyperactivity-inattention scale consisting of two items about inattention, two items about hyperactivity and one item about impulsiveness—the three symptom domains for a DSM-V diagnosis of ADHD. Each item is scored 0 to 2 thus giving a maximum symptom score of 10 with higher scores indicating greater concerns.

The Infant Characteristics Questionnaire (ICQ; Bates et al., 1979) is a short screening tool for difficultness that measures maternal perceptions of infant temperament at 6 months in four dimensions: fussy or difficult; unadaptable; dull; and unpredictable. The measure consists of 24 items rated on seven-point scales, where a rating of 1 describes an optimal temperamental trait and a rating of 7, a difficult temperament trait.

The General Health Questionnaire-28 (Sterling, 2011) is a self-reported measure used to determine if a person’s current mental state is different to usual—within BiB it was used as a measure of maternal mental health. Each question has four responses, with a Likert scoring method, where a higher score represents a worse mental state. An overall score, and four subscores can be produced: depression, anxiety/insomnia, social impairment/dysfunction and somatic symptoms.

Parenting style/practices were observed using a set of 23 self-reported questions measured at months 6 and 24—only the month 24 scores are used within this paper. Three scores can be created: warmth, hostility, and self-efficacy.

Total sleep duration was calculated as the sum of daytime and night-time hours sleep per day. This was parent reported and collected at 6, 12, 18, 24, and 36 months.

#### Statistical Analysis

All statistical analyses were undertaken using Stata version 16 (StataCorp, 2016). All analyses were pre-planned, and assessed at a 5% significance level, unless otherwise stated. The characteristics of the included infants, and their associated parents are summarized descriptively. Continuous variables are summarized using means and standard deviations, with categorical variables being described with counts and percentages.

Extreme outliers of total sleep duration were removed from the data set where a child was reported to have slept less than or equal to 4 hr in a day, or equal to or greater than 20 hr a day (*n* = 14 over the whole data set). Removal of extreme outliers had no effect on average sleep durations. At each time point the children were classified as short sleepers or normal sleepers, in respect to the sample population, using a cut off at the 25th percentile for that time point.

A key aim of this study was to explore in which window of a child’s development (0 –18 months (early) or 18 to 36 months (late)) short sleep duration had the most impact on later ADHD symptoms. It was hoped this could inform the timing of potential sleep interventions. For each window (early and late) the children were considered either a short or normal sleeper. To be classed as a short or normal sleeper in the “early” category a child was required to meet the 25th percentile criteria for two or more of the 6, 12 and, 18 months time points. To be classed as a short or normal sleeper in the “late” category a child was required to meet criteria for at least one of the 24 or 36 time points Missing data on sleep meant not all children were classified. Children were grouped into four categories:

- “early” short sleeper (0–18 months) where they had short sleep which normalized (after 18 months);
- “late” short sleeper where they had normal sleep up to 18 months but then had short sleep after 18 months;
- “consistent” short sleeper where they were classed as a short sleeper for both the early and late window;
- or a “consistent” normal sleeper, where they had normal duration of sleep throughout.

The SDQ and ICQ scores are summarized by type of sleeper, as categorized above, to allow for an informal observation of any difference between the groups.

The possibility of a relationship between early temperament (measured by the ICQ) and hyperactivity (measured by the SDQ) was explored using unadjusted linear regression, run for the SDQ at each time point using each of the components of the ICQ. This analysis was repeated, adjusted for type of sleeper, to determine if this mediated any relationship seen. Finally, the SDQ scores were compared using linear regression for those who were categorized as consistently short sleepers, with those who had early short sleepers and then improved—to explore whether an intervention at 18 months would improve ADHD symptoms.

To further explore the effect that sleep in the early years has on SDQ-measured ADHD symptoms, various covariates were explored using a linear regression model to determine their relationship to SDQ score. A *p*-value of .1 was used to determine if the covariate should be included in the multivariate model. The factors of interest were:

- Did the mother smoke during pregnancy (yes/no)
- Did the mother use drugs during pregnancy (yes/no)
- Did the mother consume alcohol during pregnancy (yes/no)
- Mother’s age at child’s birth
- Father’s age at child’s birth
- Did the child have a regular bedtime at month 24 (dichotomized as yes (5–6 times a week, or every night) and no (never, once a week, 1–3 times a week and 2–4 times a week))
- Maternal mental health (measured using the GHQ)
- Parenting practices (warmth, hostility and self-efficacy)
- Child’s temperament (ICQ—fussiness, adaptability, dullness, predictability)

### Results

The characteristics of the included 1,713 children and their parents can be seen in Table 1.

**Table 1.** Characteristics of the Included Children and Their Parents.

Characteristic	*N* = 1,713	
Sex: *n* (%)		
Female	875	(51.1)
Male	838	(48.9)
Ethnicity: *n* (%)		
British or mixed British	479	(28.0)
Pakistani or British Pakistani	721	(42.1)
Bangladeshi or British Bangladeshi	26	(1.5)
Indian or British Indian	56	(3.3)
Other Asian background	22	(1.3)
White and Black Caribbean	6	(0.4)
Other White background	25	(1.5)
White and Asian	12	(0.7)
African	21	(1.2)
Caribbean	2	(0.1)
White and Black African	2	(0.1)
Other Black background	2	(0.1)
Chinese	0	(0.0)
Irish	1	(0.1)
Other mixed background	4	(0.2)
Other	2	(0.1)
Missing	326	(19.1)
Mothers highest qualification: *n* (%)^a^		
< 5 GCSEs	376	(22.0)
5+ GCSEs	556	(32.5)
A Levels	233	(13.6)
Higher than A Levels	405	(23.6)
Missing	143	(8.4)
Fathers highest qualification: *n* (%)^a^		
<5 GCSEs	252	(14.7)
5+ GCSEs	402	(23.5)
A Levels	167	(9.8)
Higher than A Levels	433	(25.3)
Missing	459	(26.8)
Mothers age (*n* = 1713)		
Mea *n* (SD)	27.4	(5.6)
Fathers age (*n* = 610)		
Mean (SD)	30.0	(6.5)

*Note*. GCSEs typically undertaken at 16 years, A-levels at 18 years.

a UK qualifications.

### Total Sleep

**Figure 1.**
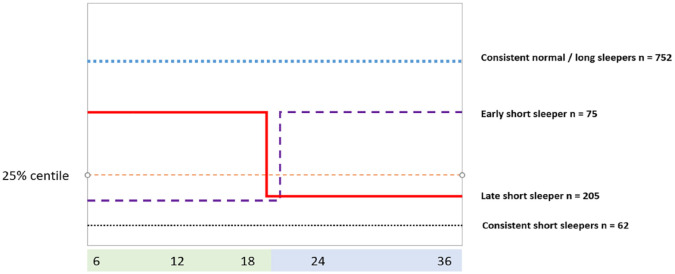
Overview of the four sleeper categories.

Details on the amount of sleep at each time point can be found in Table 2. Sixty-two children were identified as consistent short sleepers, 75 were early short sleepers improving after 18 months, 205 infants were initially normal sleepers but became short sleepers at 18 months (late short sleepers), and 752 were consistent normal sleepers—as shown in Figure 1.

**Table 2.** Mean Total Sleep in a 24 hr Period Measured in Hours, at Various Time Points.

Month	*N*	Mean (hours)	*SD*
6	1274	13.1	2.0
12	1262	12.9	1.7
18	1240	12.7	1.5
24	1181	12.4	1.4
36	1197	11.9	1.2

The SDQ hyperactivity-inattention average raw score was 4.7 at 37 months (*n* = 1995, SD 2.4), 4.1 at 54 months (*n* = 566, SD 2.3), and dropped to 2.7 at 61 months (*n* = 632, SD 2.7). The 61 month questionnaire was teacher completed rather than parent completed at 37 and 54 months, which may be a factor in the lower score seen. At 6 months the average scores for a child’s temperament, as measured by the ICQ were 17.0 for fussiness (SD 6.0, possible range 6–42), 10.2 for inadaptability (SD 4.7, possible range 4–28), 7.1 for dullness (SD 2.8, possible range 3–21) and 7.3 for unpredictability (SD 3.3, possible range 3–21).

These measures, broken down by type of sleeper (consistent short, early short, late short or consistently normal), are given in Table 3. At month 37 the mean ADHD symptom score is higher in the consistently short sleeper group (mean 5.6) compared with the other three groups which have similar scores (mean 4.6–4.8). However, this resolves by month 54 and month 61, where the scores are all similar between the groups. Smaller sample sizes here may influence these results. The average score for fussiness was higher in the consistently short sleeper group (18.3) compared with the consistent normal sleep group (16.8). Other elements of the ICQ were consistent across the sleeper groups.

**Table 3.** The ICQ Scale Scores at 6 Months, and SDQ Scores at 37, 54, and 61 Months, Detailed by Type of Sleeper.

	Consistent short sleeper (*n* = 62)	Early short sleeper (*n* = 75)	Late short sleeper (*n* = 205)	Consistent normal sleeper (*n* = 752)
ICQ (conducted at 6 months)				
Fussiness				
*N*	57	68	174	659
Mean	18.3	17.3	17.0	16.8
*SD*	5.1	6.1	6.1	5.9
Inadaptability				
*N*	58	69	174	662
Mean	10.6	10.8	10.4	10.0
*SD*	4.8	4.8	4.7	4.8
Dullness				
*N*	58	69	174	661
Mean	6.4	6.8	7.1	7.2
*SD*	2.7	2.4	2.6	2.8
Unpredictability				
*N*	58	70	176	664
Mean	8.1	8.1	7.3	7.2
*SD*	3.2	3.7	3.4	3.3
SDQ				
Month 37				
*N*	56	72	197	697
Mean	5.6	4.8	4.7	4.6
*SD*	2.5	2.8	2.4	2.4
Month 54				
*N*	30	34	89	310
Mean	4.0	4.5	3.9	4.1
*SD*	1.8	2.4	2.0	2.4
Month 61				
*N*	22	36	96	317
Mean	3.0	2.4	2.7	2.8
*SD*	2.4	2.8	2.7	2.9

The unadjusted regression results exploring the relationships between each ICQ element of temperament and ADHD symptoms at months 37, 54 and 61 can be seen in Table 4. These results show that fussiness reported at 6 months is associated with ADHD symptoms measured at 37 months (*p* < .00) and 54 months (*p* = .02) though not at 61 months. At both timepoints, the associated coefficient is positive, suggesting that higher levels of fussiness seen at 6 months, will result in higher levels of ADHD symptoms. No other element of the ICQ was found to be associated with SDQ hyperactivity.

**Table 4.** Unadjusted Linear Regression Results Comparing the Relationship of SDQ Hyperactivity and Each Element of the ICQ at 37, 54, and 61 Months.

	*N*	Coefficient	95% CI	*p*-value
Month 37				
ICQ: Fussiness	969	0.07	[0.05, 0.10]	<.00
ICQ: Unadaptable	976	0.03	[−0.00, 0.06]	.06
ICQ: Dull	976	−0.04	[−0.09, 0.02]	.19
ICQ: Unpredictable	981	0.03	[−0.02, 0.08]	.23
Month 54				
ICQ: Fussiness	455	0.04	[0.01, 0.08]	.02
ICQ: Unadaptable	461	0.02	[−0.03, 0.06]	.45
ICQ: Dull	461	0.02	[−0.05 , 0.10]	.58
ICQ: Unpredictable	463	0.05	[−0.01, 0.11]	.13
Month 61				
ICQ: Fussiness	507	−0.01	[−0.05, 0.03]	.57
ICQ: Unadaptable	510	−0.04	[−0.10, 0.01]	.08
ICQ: Dull	511	0.02	[−0.07, 0.10]	.73
ICQ: Unpredictable	515	−0.04	[−0.11, 0.03]	.28

Repeating the above analysis, with the inclusion of sleeper type did not modify the results. There was still a significant association between fussiness and ADHD symptoms at months 37 and 54, and no other significant association present (Table 5). This suggests that sleeper type—that is a short sleeper in the early or later years—does not mediate the relationship between child temperament and ADHD symptoms as measured by the SDQ.

**Table 5.** Linear Regression Results Comparing SDQ Hyperactivity at 37, 54, and 61 Months With Each Element of the ICQ Adjusted by Type of Sleeper.

	*N*	Coefficient	95% CI	*p*-value
Month 37				
ICQ: Fussiness	901	0.07	[0.05, –0.10]	<.00
ICQ: Unadaptable	906	0.03	[−0.00, 0.07]	.06
ICQ: Dull	906	−0.05	[−0.10, 0.01]	.13
ICQ: Unpredictable	911	0.03	[−0.02, 0.08]	.27
Month 54				
ICQ: Fussiness	409	0.05	[0.01, 0.09]	.01
ICQ: Unadaptable	414	0.01	[−0.04, 0.06]	.68
ICQ: Dull	414	0.01	[−0.07, 0.09]	.76
ICQ: Unpredictable	415	0.03	[−0.03, 0.10]	.35
Month 61				
ICQ: Fussiness	423	−0.01	[−0.06, 0.03]	.55
ICQ: Unadaptable	425	−0.05	[−0.11, 0.01]	.08
ICQ: Dull	426	−0.01	[−0.11, 0.09]	.83
ICQ: Unpredictable	430	−0.06	[−0.14, 0.02]	.12

A comparison of ADHD symptoms between the subgroup of infants with consistent short sleep and those with short sleep until month 18 which then improves, using a linear regression, can be seen in Table 6. There are no statistically significant results seen here, suggesting that an intervention aimed at improving length of sleep after 18 months may not lead to a reduction in ADHD symptoms. However, the sample size for these analyses was small, limiting the reliability of the results.

**Table 6.** Linear Regression Results Comparing the SDQ Score for Consistently Short Sleepers With Those Who are Early Short Sleepers.

	*N*	Coefficient	95% CI	*p*-value
Month 37	128	−0.78	[−1.73, 0.17]	.11
Month 54	64	0.50	[−0.58, 1.58]	.36
Month 61	58	−0.66	[−2.10, 0.79]	.37

The results of exploring the relationship of parent/child characteristics on the SDQ at month 37 are summarized in Table 7.

**Table 7.** Results of the Exploration of Parent/Child Characteristics With Hyperactivity Measured by the SDQ at M37 for Inclusion With the Multivariate Model.

Factor	*N*	*p*-value	Included within model
Smoking (yes/no)	266	.82	No
Drug use yes/no)	1193	.48	No
Alcohol use (yes/no)	164	.68	No
Mother’s age	1195	<.00	Yes
Father’s age	447	.03	No^b^
Regular bedtime at M24 (yes/no)	1092	<.00	Yes
GHQ: Somatic Symptoms	960	<.00	No^a^
GHQ: Anxiety/Insomnia	947	.02	No^a^
GHQ: Social Dysfunction	973	.34	No^a^
GHQ: Depression	961	.06	No^a^
GHQ: Total Score	917	<.00	Yes^a^
Gender (male/female)	1195	<.00	Yes
Parenting Style: Warmth M24	1084	.03	Yes
Parenting Style: Hostility M24	1093	<.00	Yes
Parenting Style: Self-efficacy M24	1087	<.00	Yes
ICQ: Fussiness	969	<.00	Yes
ICQ: Unadaptable	976	.06	Yes
ICQ: Dull	976	.19	No
ICQ: Unpredictable	981	.23	No

a As three of the four subscales of the GHQ and total score were found to be significant, only the total score will be included, to reduce the number of covariates included in the model.

b Due to the limited data on age of the father, and to increase the number of children in the model—father’s age was omitted.

The multivariate model showed that, after adjusting for other factors that may influence the SDQ score, both late short sleepers and consistently normal sleepers (i.e., those who slept well in the first 18 months of their lives), had a lower SDQ at month 37 compared to consistent short sleepers (coeff. −0.84, 95% CI [−1.61, −0.07], *p* = .03 for late short sleepers; coeff. −0.75, 95% CI [−1.45, −0.06], *p* = .03 for consistent normal sleepers). However, this was not true for the early short sleepers (coeff. −0.51, 95% CI [−1.42, 0.40], *p* = .27). This suggests, perhaps, that sleep in the first 18 months of a child’s life is symptomatic of their ADHD symptoms at month 37.

When running the same model for SDQ scores at months 54 and 61, none of the sleeper categories were found to have a SDQ score statistically different to that of the consistent short sleepers (*p* = .39, 0.73 and 0.67 at M54 and *p* = .94, 0.74 and 0.83 at month 61, for early short, late short and consistently normal respectively).

## Discussion

This study explored the association between sleep duration, temperament and symptoms of Attention Deficit Hyperactivity Disorder (ADHD) in a biethnic population of children using parent and teacher reported measures.

Children with normal sleep before 18 months had fewer ADHD symptoms at 37 months compared to those with consistently short sleep. One dimension of temperament (fussiness) reported by parents at 6 months of age was found to be significantly positively associated with hyperactivity and inattention symptoms measured at 37 months and 54 months. No other dimension of temperament was found to be significantly associated with ADHD symptoms at any time point. Fussiness was not significantly different between the categories of sleeper and it does not appear to mediate the relationship between sleep duration and ADHD symptoms. Additionally, sleep duration was not found to mediate the relationship between fussiness and ADHD symptoms.

These results support our first hypothesis that sleep duration and infant temperament would be associated with ADHD symptoms. However, the results do not support our second hypothesis that the relationship between sleep duration and ADHD symptoms would be mediated by infant temperament.

Overall these findings suggest infant fussiness is an early indicator of risk for later childhood ADHD symptoms. An evident association so early in childhood lends weight to inherited neuro-biological dysfunction in the frontal brain accounting for both early short sleep duration and inattentive symptoms.

This result complements the finding of Miller et al. (2019) which found boys’ infant motor activity was positively predictive of later ADHD symptoms though only with lower quality maternal caregiving behaviors. For girls motor activity was negatively predictive of later ADHD symptoms though with high quality maternal caregiving behaviors.

Sub-categorization of patterns of sleep duration allowed a detailed exploration of potential sleep sensitive windows of development. We hoped this may inform the possible benefits of sleep interventions in early childhood in the mitigation of later ADHD symptoms. Eighteen months was chosen as the point to dichotomize sleep problems into early and late categories as it was felt this was the earliest practical time infant sleep problems could be recognized and brought to the attention of a clinician. After adjusting for other factors which may influence the ADHD symptoms, we found that children with normal sleep duration in their first 18 months whose sleep duration shortens from 18 to 36 months have significantly lower ADHD symptom scores (coefficient −0.84, *p* = .03) at month 37 when compared with consistently short sleepers, as did those who consistently had normal sleep (coefficient −.75, *p* = .03). These results suggest sleep duration in the first 18 months is of more importance in the etiology of ADHD than sleep duration from 18 to 36 months. Significance is not carried forward for ADHD symptom scores measured at months 54 and 61, however the samples at these time points are reduced.

Our main finding is consistent with other prospective cohort studies which show associations between sleep problems and externalizing symptoms (Bron et al., 2016; Scott et al., 2013; E. Touchette et al., 2009). However, where many previous studies are limited by a lack of measures of sleep duration in early childhood, this study’s measure of sleep duration at 6 and 12 months allows greater exploration of the role of sleep duration during this critical development period in early infancy. Our main finding is in line with other longitudinal sleep studies conducted from early infancy. Sleep duration at 3, 8 and 24 months was found to be associated with inattentiveness in 5 years old children as measured by the SDQ in early childhood (Huhdanpää et al., 2019). A study of sleep problem trajectories spanning ages 0 to 1 to 10 to 11 found persistent sleep problems were associated with multiple impairments in wellbeing including large carer-giver reported externalizing concerns again measured by the SDQ (Williamson et al., 2020). Importantly our study is the first to demonstrate that such associations hold true in a biethnic population with a majority South Asian ethnicity (48.2%).

### Strengths

This study has several strengths. Multiple measures of sleep duration across infancy is not common among studies in this field. Our study captures the continued impact of sleep duration over time on ADHD symptoms. Categorization of sleep patterns facilitated subgroup analysis of the effect of persistent sleep patterns on later ADHD symptoms. Furthermore few studies include measures of sleep duration in early infancy which provided an opportunity to explore early altered developmental pathways of sleep quantity.

This longitudinal study adds to the body of evidence in this field, in part addressing the lack of cohort studies highlighted in a recent systematic review (Lee et al., 2019). Its comparatively large size (*n* = 1713) allowed an exploration of causality with parent reported fussiness acting as a proxy baseline measure of ADHD symptoms. As sleep type did not modify the relationship between early fussiness and later ADHD symptoms this may suggest sleep duration is not a contributor to ADHD symptoms but another consequence of inherited neuro-biological dysfunction. Although conclusions are tentative, the use of infant temperament at 6 months in a longitudinal study is novel.

Few sleep duration/ADHD studies have been conducted within ethnically diverse populations nor populations with significant Asian ethnic origin proportion (Touchette, 2009 92.1% white, Scott et al., 2013, only non-white category). Equally most studies have been conducted in Europe with few studies conducted in Asia.

### Limitations

A limitation of longitudinal studies that run over multiple years is missing data. Sample sizes for sleep duration at 54 and 61 months (*n* = 462 and 514 respectively) were approximately half that at 37 months (*n* = 981). Loss to follow-up is most likely in socioeconomic deprived groups and younger families (Scott et al., 2013), both groups previously shown to have higher incidences of children with ADHD symptoms.

Measures of sleep duration and ADHD symptoms were subjectively reported by parents and teachers in this study. We attempted to explore the potential bias and bidirectional nature of the relationship between children’s sleep and parental mental health with use of the GHQ-28. However, objective measures such as actigraphy or polysomnography would provide a preferable measure of sleep duration; however this can place a high burden on the families and may influence parental behaviors when they are aware their child’s sleep is being monitored. Furthermore, the highly familial nature of ADHD would suggest parents reporting their children’s ADHD symptoms are themselves more likely to experience inattentive symptoms. This could lead to greater reporting errors in this group.

ADHD symptoms were measured through parent and teacher reported SDQ. We recognize that diagnosis by an experienced child psychiatrist in line with current ICD11 criteria is preferable though use of SDQ is a prevailing outcome measure in recent longitudinal studies in this field (Huhdanpää et al., 2019; Peralta et al., 2018; Williamson et al., 2020). The 61 months SDQ was teacher-reported unlike 37 and 54 months parent-reported SDQ scores. Children at 61 months are in their first year at school where impulsive, inattentive symptoms may be more challenging to identify as all children learn to adjust to the new classroom environment and associated rules.

Sub-group analysis requiring the classification of children into types of sleeper led to groups that varied considerably in size. With some children not fitting into specific predefined categories (*n* = 619 uncategorized, 36% of the sample), and the consistent short sleeper group was small (*n* = 62) reducing the power of this specific analysis.

Equally the data within this study did not utilize information regarding variability of sleep duration, children’s bedtime routine, or nighttime awakenings.

At the time of analysis no data was available beyond 61 months as the cohort study was ongoing. This may be released at a future point in time.

## Conclusions and Future Research

In conclusion, our study found children with normal sleep duration in the first 18 months (both those who had consistently normal sleep, and those who had normal sleep that was short at 18–36 months), have significantly lower ADHD symptom scores measured at 37 months than infants with consistent short sleep durations. We also found fussiness of a child early in its life at 6 months was significantly associated with hyperactivity and inattention symptoms measured at 37 months and 54 months. Sleep duration patterns in early childhood appeared not to modify this relationship. Together these findings tentatively support a theory of inherited neuro-biological dysfunction accounting for both early short sleep duration and hyperactive/inattentive symptoms.

The finding that fussiness at 6 months is predictive of later ADHD symptoms at 37 months provides an early indicator of potential future difficulties which may be utilized to aid earlier diagnosis.

As the Born in Bradford study progresses we may be provided with future opportunities to identify which children receive ADHD diagnoses and whether relationships with sleep exist in later childhood.

## References

[bibr1-10870547231168433] Algorta G. P. Dodd A. L. Stringaris A. Youngstrom E. A. (2016). Diagnostic efficiency of the SDQ for parents to identify ADHD in the UK: A ROC analysis. European Child & Adolescent Psychiatry, 25, 949–957. 26762184 10.1007/s00787-015-0815-0 PMC4990620

[bibr2-10870547231168433] Bates J. E. Freeland C. A. Lounsbury M. L. (1979). Measurement of infant difficultness. Child Development, 50, 794–803. 498854

[bibr3-10870547231168433] Bron T. I. Bijlenga D. Kooij J. J. Vogel S. W. Wynchank D. Beekman A. T. Penninx B. W. (2016). Attention-deficit hyperactivity disorder symptoms add risk to circadian rhythm sleep problems in depression and anxiety. Journal of Affective Disorders, 200, 74–81. 27128360 10.1016/j.jad.2016.04.022

[bibr4-10870547231168433] Chung W. Jiang S. F. Paksarian D. Nikolaidis A. Castellanos F. X. Merikangas K. R. Milham M. P. (2019). Trends in the prevalence and incidence of attention-deficit/hyperactivity disorder among adults and children of different racial and ethnic groups. JAMA network open, 2(11), e1914344. 31675080 10.1001/jamanetworkopen.2019.14344 PMC6826640

[bibr5-10870547231168433] DeGangi G. A. Breinbauer C. Roosevelt J. D. Porges S. Greenspan S. (2000). Prediction of childhood problems at three years in children experiencing disorders of regulation during infancy. Infant Mental Health Journal, 21(3), 156–175.

[bibr6-10870547231168433] Degangi G. A. Dipietro J. A. Greenspan S. I. Porges S. W. (1991). Psychophysiological characteristics of the regulatory disordered infant. Infant Behavior and Development, 14(1), 37–50.

[bibr7-10870547231168433] DeSantis A. Coster W. Bigsby R. Lester B. (2004). Colic and fussing in infancy, and sensory processing at 3 to 8 years of age. Infant Mental Health Journal, 25(6), 522–539.

[bibr8-10870547231168433] Efron D. (2019). Attention deficit hyperactivity disorder: An overview. Sleep and ADHD, 1, 1–28.

[bibr9-10870547231168433] Fairman K. A. Peckham A. M. Sclar D. A. (2020). Diagnosis and treatment of ADHD in the United States: Update by gender and race. Journal of Attention Disorders, 24(1), 10–19. 28152660 10.1177/1087054716688534

[bibr10-10870547231168433] Fayyad J. Sampson N. A. Hwang I. Adamowski T. Aguilar-Gaxiola S. Al-Hamzawi A. Kessler R. C. (2017). The descriptive epidemiology of DSM-IV adult ADHD in the world health organization world mental health surveys. ADHD Attention Deficit and Hyperactivity Disorders, 9(1), 47–65. 27866355 10.1007/s12402-016-0208-3 PMC5325787

[bibr11-10870547231168433] Ford T. Goodman R. Meltzer H. (2003). The British child and adolescent mental health survey 1999: The prevalence of DSM-IV disorders. Journal of the American Academy of Child and Adolescent Psychiatry, 42(10), 1203–1211. 14560170 10.1097/00004583-200310000-00011

[bibr12-10870547231168433] Forsyth B. W. Canny P. F. (1991). Perceptions of vulnerability 3½ years after problems of feeding and crying behaviour in early infancy. Pediatrics, 88(4), 757–763. 1896279

[bibr13-10870547231168433] Hall C. L. Guo B. Valentine A. Z. Groom M. J. Daley D. Sayal K. Hollis C. (2019). The validity of the Strengths and Difficulties Questionnaire (SDQ) for children with ADHD symptoms. PLoS One, 14(6), e0218518. 31216327 10.1371/journal.pone.0218518 PMC6583960

[bibr14-10870547231168433] Hemmi M. H. Wolke D. Schneider S. (2011). Associations between problems with crying, sleeping and/or feeding in infancy and long-term behavioural outcomes in childhood: A meta-analysis. Archives of Disease in Childhood, 96, 622–629. 21508059 10.1136/adc.2010.191312

[bibr15-10870547231168433] Holden S. E. Jenkins-Jones S. Poole C. D. Morgan C. L. Coghill D. Currie C. J. (2013). The prevalence and incidence, resource use and financial costs of treating people with attention deficit/hyperactivity disorder (ADHD) in the United Kingdom (1998 to 2010). Child and Adolescent Psychiatry and Mental Health, 7(1), 1–13. 24119376 10.1186/1753-2000-7-34 PMC3856565

[bibr16-10870547231168433] Huhdanpää H. Morales-Muñoz I. Aronen E. T. Pölkki P. Saarenpää-Heikkilä O. Paunio T. Kylliäinen A. Paavonen E. J. (2019). Sleep difficulties in infancy are associated with symptoms of inattention and hyperactivity at the age of 5 years: A longitudinal study. Journal of Developmental & Behavioral Pediatrics, 40(6), 432–440. 31166249 10.1097/DBP.0000000000000684 PMC6738636

[bibr17-10870547231168433] Khan T. A. Hussain S. Ikram A. Mahmood S. Riaz H. Jamil A. Amin A. Haider Y. G. Sandhu M. Mushtaq A. Barbui C. Johnson C. F. Godman B. (2020). Prevalence and treatment of neurological and psychiatric disorders among tertiary hospitals in Pakistan; findings and implications. Hospital Practice, 48(3), 145–160. 32343632 10.1080/21548331.2020.1762366

[bibr18-10870547231168433] Lee S. H. Kim H. B. Lee K. W. (2019). Association between sleep duration and attention-deficit hyperactivity disorder: A systematic review and meta-analysis of observational studies(✰). Journal of Affective Disorders, 256, 62–69. 31158717 10.1016/j.jad.2019.05.071

[bibr19-10870547231168433] Melegari M. G. Vittori E. Mallia L. Devoto A. Lucidi F. Ferri R. Bruni O. (2020). Actigraphic sleep pattern of preschoolers with ADHD. Journal of Attention Disorders, 24(4), 611–624. 27708108 10.1177/1087054716672336

[bibr20-10870547231168433] Miller N. V. Degnan K. A. Hane A. A. Fox N. A. Chronis-Tuscano A. (2019). Infant temperament reactivity and early maternal caregiving: independent and interactive links to later childhood attention-deficit/hyperactivity disorder symptoms. Journal of Child Psychology and Psychiatry, 60(1), 43–53. 29889314 10.1111/jcpp.12934 PMC6289898

[bibr21-10870547231168433] Nøvik T. S. Hervas A. Dalsgaard S. Rodrigues Pereira R. Lorenzo M. J. (2006). Influence of gender on attention-deficit/hyperactivity disorder in Europe–ADORE. European Child & Adolescent Psychiatry, 15(1), i15–i24. 17177011 10.1007/s00787-006-1003-z

[bibr22-10870547231168433] Owens J. A. (2005). The ADHD and sleep conundrum: a review. J. Dev. Behav. Pediatr., 26, 312–322. 16100507 10.1097/00004703-200508000-00011

[bibr23-10870547231168433] Peralta G. P. Forns J. García de la Hera M. González L. Guxens M. López-Vicente M. Sunyer J. Garcia-Aymerich J. (2018). Sleeping, TV, cognitively stimulating activities, physical activity, and attention-deficit hyperactivity disorder symptom incidence in children: A prospective study. Journal of Developmental & Behavioral Pediatrics, 39(3), 192–199. 29261536 10.1097/DBP.0000000000000539

[bibr24-10870547231168433] Scher A. (2005). Infant sleep at 10 months of age as a window to cognitive development. Early Human Development, 81(3), 289–292. 15814211 10.1016/j.earlhumdev.2004.07.005

[bibr25-10870547231168433] Scott N. Blair P. S. Emond A. M. Fleming P. J. Humphreys J. S. Henderson J. Gringras P. (2013). Sleep patterns in children with ADHD: A population-based cohort study from birth to 11 years. Journal of Sleep Research, 22, 121–128. 23057438 10.1111/j.1365-2869.2012.01054.x PMC3786528

[bibr26-10870547231168433] Spruyt K. Gozal D. (2011). Sleep disturbances in children with attention-deficit/hyperactivity disorder. Expert Review of Neurotherapeutics, 11(4), 565–577. 21469929 10.1586/ern.11.7 PMC3129712

[bibr27-10870547231168433] StataCorp. (2016). Stata Statistical Software: Release 16. College Station, TX: StataCorp LLC.

[bibr28-10870547231168433] Sterling M. (2011). General Health Questionnaire - 28 (GHQ-28). Journal of Physiotherapy, 57(4), 259. 22093128 10.1016/S1836-9553(11)70060-1

[bibr29-10870547231168433] Sullivan E. L. Holton K. F. Nousen E. K. Barling A. N. Sullivan C. A. Propper C. B. Nigg J. T. (2015). Early identification of ADHD risk via infant temperament and emotion regulation: A pilot study. Journal of Child Psychology and Psychiatry, 56(9), 949–957. 25968589 10.1111/jcpp.12426

[bibr30-10870547231168433] Sung V. Hiscock H. Sciberras E. Efron D. (2008). Sleep problems in children with attention-deficit/hyperactivity disorder: Prevalence and the effect on the child and family. Archives of Pediatrics & Adolescent Medicine, 162(4), 336–342. 18391142 10.1001/archpedi.162.4.336

[bibr31-10870547231168433] Touchette E. Côté S. M. Petit D. Liu X. Boivin M. Falissard B. Tremblay R. E. Montplaisir J. Y. (2009). Short nighttime sleep-duration and hyperactivity trajectories in early childhood. Pediatrics, 124(5), e985–e993. 19841107 10.1542/peds.2008-2005

[bibr32-10870547231168433] Ullebø A. K. Posserud M. B. Heiervang E. Gillberg C. Obel C. (2011). Screening for the attention deficit hyperactivity disorder phenotype using the strength and difficulties questionnaire. European Child & Adolescent Psychiatry, 20, 451–458. 21833627 10.1007/s00787-011-0198-9

[bibr33-10870547231168433] Willcutt E. G. (2012). The prevalence of DSM-IV attention-deficit/hyperactivity disorder: a meta-analytic review. Neurotherapeutics, 9(3), 490–499. 22976615 10.1007/s13311-012-0135-8 PMC3441936

[bibr34-10870547231168433] Williamson A. A. Mindell J. A. Hiscock H. Quach J. (2020). Longitudinal sleep problem trajectories are associated with multiple impairments in child well-being. Journal of Child Psychology and Psychiatry, 61(10), 1092–1103. 32713013 10.1111/jcpp.13303 PMC7530051

[bibr35-10870547231168433] Wolke D. Meyer R. Ohrt B. Riegel K. (1995). Co-morbidity of crying and feeding problems with sleeping problems in infancy: Concurrent and predictive associations. Early Development and Parenting, 4(4), 191–207.

[bibr36-10870547231168433] Wolke D. Rizzo P. Woods S. (2002). Persistent infant crying and hyperactivity problems in middle childhood. Pediatrics, 109(6), 1054–1060. 12042542 10.1542/peds.109.6.1054

[bibr37-10870547231168433] Wright N. Moldavsky M. Schneider J. Chakrabarti I. Coates J. Daley D. Kochhar P. Mills J. Sorour W. Sayal K. (2015). Practitioner review: pathways to care for ADHD–a systematic review of barriers and facilitators. Journal of Child Psychology and Psychiatry, 56(6), 598–617. 25706049 10.1111/jcpp.12398 PMC5008177

[bibr38-10870547231168433] Yoon S. Y. Jain U. Shapiro C. (2012). Sleep in attention-deficit/hyperactivity disorder in children and adults: Past, present, and future. Sleep Medicine Reviews, 16(4), 371–388. 22033171 10.1016/j.smrv.2011.07.001

[bibr39-10870547231168433] Yürümez E. Kılıç B. G. (2016). Relationship between sleep problems and quality of life in children with ADHD. Journal of Attention Disorders, 20(1), 34–40. 23511553 10.1177/1087054713479666

[bibr40-10870547231168433] Zero to Three. (2005). DC:0-3R: Diagnostic classification of mental health and developmental disorders of infancy and early childhood (Rev. ed.). ZERO TO THREE/National Center for Infants, Toddlers and Families.

